# Recent advances toward understanding the role of transplanted stem cells in tissue-engineered regeneration of musculoskeletal tissues

**DOI:** 10.12688/f1000research.21333.1

**Published:** 2020-02-18

**Authors:** Dallas E. Altamirano, Kathleen Noller, Eszter Mihaly, Warren L. Grayson

**Affiliations:** 1 Department of Biomedical Engineering, Johns Hopkins University School of Medicine, Baltimore, MD, 21205, USA; 2 Translational Tissue Engineering Center, Johns Hopkins University School of Medicine, Baltimore, MD, 21231, USA; 3 Duke University Medical School, Duke University, Durham, NC, 27710, USA; 4 Department of Materials Science & Engineering, Johns Hopkins University School of Engineering, Baltimore, MD, 21231, USA; 5 Institute for NanoBioTechnology, Johns Hopkins University School of Engineering, Baltimore, MD, 21231, USA

**Keywords:** Musculoskeletal tissue engineering, stem cells

## Abstract

Stem cell–based tissue engineering is poised to revolutionize the treatment of musculoskeletal injuries. However, in order to overcome scientific, practical, and regulatory obstacles and optimize therapeutic strategies, it is essential to better understand the mechanisms underlying the pro-regenerative effects of stem cells. There has been an attempted paradigm shift within the last decade to think of transplanted stem cells as “medicinal” therapies that orchestrate healing on the basis of their secretome and immunomodulatory profiles rather than acting as *bona fide* stem cells that proliferate, differentiate, and directly produce matrix to form *de novo* tissues. Yet the majority of current bone and skeletal muscle tissue engineering strategies are still premised on a direct contribution of stem cells as building blocks to tissue regeneration. Our review of the recent literature finds that researchers continue to focus on the quantification of *de novo* bone/skeletal muscle tissue following treatment and few studies aim to address this mechanistic conundrum directly. The dichotomy of thought is reflected in the diversity of new advances ranging from *in situ* three-dimensional bioprinting to a focus on exosomes and extracellular vesicles. However, recent findings elucidating the role of the immune system in tissue regeneration combined with novel imaging platform technologies will have a profound impact on our future understanding of how stem cells promote healing following biomaterial-mediated delivery to defect sites.

## Introduction

Bone and skeletal muscle both have high self-healing capacities that become overwhelmed when loss of large tissue volumes results in critical-sized bone defects or volumetric muscle loss (VML). Current pre-clinical tissue engineering strategies for musculoskeletal defects employ bone marrow–derived mesenchymal stem cells (MSCs), adipose-derived stromal/stem cells (ASCs), other tissue-derived stem cell populations, and induced pluripotent stem cells (iPSCs). However, although there are over 200 clinical trials involving ASCs and MSCs in the US ( www.clinicaltrials.gov), very few of these trials involve tissue engineering strategies to regenerate musculoskeletal tissues. There are considerable scientific, practical, and regulatory hurdles that so far have restricted the translational use of stem cells for these applications. Underlying all of these hurdles is a dearth of understanding of the mechanism by which transplanted cells promote regeneration. This has imposed significant limitations on our ability to optimize key parameters such as stem cell purity, dosing, priming, delivery, or the design of scaffold biomaterials. In fact, the majority of clinical trials are not using ASCs/MSC for direct differentiation and contribution to tissue repair but rely on paracrine mechanisms. Yet few recent studies have focused on improving our understanding of the fate of transplanted cells. In spite of this, a convergence of stem cell–based tissue engineering with recent advances in imaging technologies and immunoengineering may open a window of opportunity for further exploration and the discovery of new insights.

## Transplanted stem cells orchestrate musculoskeletal tissue repair

### Bone tissue engineering

The current gold standard of treatment of the critical-sized bone defect is the cancellous autograft, often harvested from the iliac crest ^1^. Autografts repair bone via osseointegration and osteoconduction, but the use of these grafts is limited by donor site morbidity, risk of infection, risk of surgical complications, and limited available bone volume ^2^; thus, tissue engineering provides a promising alternative. Most musculoskeletal tissue engineering studies operate on the premise that stem cells seeded into biomaterial scaffolds and implanted into volumetric defects survive the ensuing ischemic microenvironment, differentiate into osteoblasts or myocytes, and integrate with the native matrix to directly impact tissue regeneration ^3^. Hence, technologies such as *in situ* bioprinting are continually being advanced to precisely control the spatial location of cells and assess the impact of different “geometries” ^4^. Other recent studies to achieve bone healing examined the effect of stimulating endochondral ossification with ASCs differentiated into hypertrophic chondrocytes and implanted into a rat femoral defect. They hypothesize that implanted hypertrophic chondrocytes help to both regulate endogenous cell behavior and directly contribute to bone formation; however, they did not determine whether improved bone formation was predominantly due to the survival and integration of the implanted cells or to their superior secretory and immunomodulatory properties ^5^. A study by Larson *et al*. demonstrated that contact with viable bone shifted the phenotype of chondrogenically pre-cultured MSCs to hypertrophic and osteogenic phenotypes in three-dimensional (3D) cultures of MSCs and in a nude rat model ^6^. Implanted chondrogenically pre-cultured MSCs, but not non-differentiated MSCs, seeded on polycaprolactone (PCL) scaffolds exhibited mineralization, formation of trabecula-like structures, and chondrogenic and osteogenic gene expression profiles at 8 weeks. Interestingly, scaffolds seeded with chondrogenically pre-cultured MSCs were the only group with human DNA present at 8 weeks and with 45% human RNA content. The findings from these studies suggest that the direct contribution of transplanted cells to bone regeneration might depend heavily on the pre-implantation priming ^5, 6^.

### Skeletal muscle tissue engineering

A variety of stem cell types have been explored as a potential source of myogenic cell replacement therapy. ASCs, which provide a high rate of stem cell proliferation and can potentially be sourced directly from the patient, bypassing immune rejection, have been evaluated extensively in the treatment of musculoskeletal damage ^7– 12^. However, recent studies have called into question whether ASC-derived cells are contributing to *de novo* myofiber regeneration directly: Gorecka *et al*. injected autologous ASCs into the tibialis anterior (TA) of mice following a crush injury and found that while the cell transplantation resulted in increased fiber cross-sectional area and improved muscle contractility, ASC-derived cells did not differentiate into myofibers or fuse with endogenous muscle fibers ^13^. Similarly, Gilbert-Honick *et al*. seeded ASCs onto electrospun fibrin hydrogels and transplanted this construct into a mouse model of VML injury ^14^. Although there was an increase in fiber cross-sectional area, only limited expression of myogenic markers in the donor cells was observed. Alternative mechanisms for the therapeutic benefit of ASC transplantation have been posited. Secretion of paracrine factors by transplanted stem cells may improve regeneration by activating endogenous muscle stem cells, by reducing inflammation at the site of the injury, or by promoting angiogenesis ^15– 17^. In particular, a number of recent studies have documented anti-fibrotic effects of transplanted stem cells. Di Summa *et al*. found that differentiated ASCs incorporated into fibrin nerve conduits and transplanted into a rat nerve gap model reduced fibrotic tissue formation, enhancing axonal regeneration and remyelination ^18^. Milosavljevic *et al*. injected MSCs or MSC-conditioned medium intravenously into mice and demonstrated that CCl4-induced liver fibrosis was attenuated ^19^. They found that MSCs acted on fibrosis by means of decreasing levels of inflammatory T helper 17 (Th17) cells while increasing anti-inflammatory CD4 ^+^ interleukin 10–positive (IL-10 ^+^) T cells. These anti-fibrotic effects of ASCs and MSCs may play a role in their regenerative capabilities upon transplantation into injury models.

A number of recent studies have explored the use of human iPSCs as a source of myogenic cell transplantations. Multiple groups have demonstrated that iPSC-derived myogenic cells contribute directly to the formation of new myofibers in damaged tissue ^20– 22^. Rao *et al*. generated the first 3D contractile skeletal muscle constructs from human iPSCs ^23^. After transplantation, the cells formed densely packed, aligned myofibers and retained functional responses. Wu *et al*. injected iPSC-derived myogenic progenitors into cardiotoxin-injured mouse TA and observed engraftment and contribution of the iPSC-derived cells to new myofiber formation ^24^. However, long-term survival of iPSC-derived cells in the transplanted environment is a limitation, multiple groups have reported high levels of cell death upon transplantation ^21, 23^, and further work is necessary to characterize the therapeutic mechanism of transplanted iPSC-derived cells. An emergent alternative to the treatment of VML is the use of autologous minced muscle grafts, or 1 mm ^3^ pieces of muscle, which have also been demonstrated to attenuate T lymphocyte and macrophage responses to severe muscle injury. These results may indicate a promising therapeutic role for cell aggregates and immunomodulatory therapies in the treatment of VML ^25^.

## Modulating the survival and secretory profile of transplanted stem cells

Tissue engineering studies continue to prioritize the “direct contribution” paradigm. They focus largely on the quantification of *de novo* bone/skeletal muscle tissue and the positive effects of stem cell delivery. However, most studies do not track implanted cells *in vivo* or quantify their viability over a long time course. Multiple studies that have tracked transplanted cells have demonstrated that few cells survive more than 4 to 8 weeks following transplantation, suggesting that their pro-regenerative outcomes might be more correctly attributed to indirect mechanisms such as cytokine secretion, immunomodulation, and signaling to endogenous cells ^26, 27^. For example, a recent study in which ASCs were injected systemically or locally into a wound bed showed that systemically delivered ASCs became trapped in the lung and could not be detected 72 hours after systemic injection but that locally injected cells remained strongly detectable up to 7 days at the wound site, yet both groups exhibited enhanced wound healing ^28^. Recently, some groups have investigated stem cell aggregation and its impact on metabolic and secretory profiles while other studies of exosomes and extracellular vesicles (EVs) have been performed in an effort to provide greater insights into the potency of transplanted stem cells.

### Stem cell aggregates

Implanting stem cells as aggregates rather than monodispersed cells has been shown to enhance their viability, migration, and differentiation and modifies their secretion of cytokines, immunomodulatory factors, and EVs to improve therapeutic outcomes ^29, 30^. Yet the lack of standardization renders it impossible to accurately correlate the impact of aggregate sizes, methods of aggregation, and mode of implantation on the therapeutic outcomes. Recent investigations into bone tissue engineering have used periosteum-derived stem cells embedded in collagen type 1 hydrogel ^31^, MSCs embedded in a platelet-rich plasma construct ^32^, or arginine-glycine-aspartic acid (RGD) functionalized alginate ^33^. The studies employed 250 cells per aggregate, randomly sized spontaneous aggregates, and 500 cells per aggregate, respectively. Aggregation increased osteogenic and chondrogenic markers and paracrine secretions. When compared with monodispersed cell-laden counterparts, aggregate-laden scaffolds showed increased bone formation ^32^ but no beneficial impact on cell survival or construct vascularization when implanted subcutaneously ^31^. However, when stem cell aggregates were used in conjunction with bone morphogenetic protein 2 (BMP2) stimulation, there was increased blood vessel formation, BMP2 production, presence of hypertrophic chondrocytes, and remodeling ^31^, although there was no increase in bone volume or torsional strength of the resulting bone ^33^. For skeletal muscle tissue engineering, recent studies have tested human umbilical cord–derived MSCs ^34^ as aggregate sheets encasing porcine heart decellularized extracellular matrix and green fluorescent protein (GFP)-labeled murine MSCs ^35^ (500 cells per aggregate) injected in phosphate-buffered saline, respectively. In both cases, the investigators observed increased recovery and peak isometric torque observed from aggregates compared with single cells ^35^.

### Extracellular vesicles/exosomes

EVs such as exosomes or microvesicles carry important cargo for cell communication and are influential in cell signaling. Exosomes and microvesicles are distinguished by their sizes and origins (that is, endocytic pathway versus plasma membranes). They both contain lipids, nucleic acids, and protein cargo. Studies have shown that EVs can enhance cell differentiation and viability, which in turn may affect therapeutic outcomes, although the molecular underpinnings of this are not yet understood. It has recently been suggested that EVs are heavily involved in bone homeostasis ^36^ and skeletal muscle myogenesis ^37^, but further investigations are necessary in order to elucidate the mechanisms in which these occur. For bone tissue engineering, recent studies of MSC-derived EVs or exosomes embedded in a hydrogel for treating critical-sized calvarial defect have demonstrated therapeutic benefit with significant increases in bone volume fraction, bone mineral density, and new bone area ^38– 40^. Furthermore, combining EVs with MSCs resulted in increased bone volume and bone volume fraction compared with either component delivered separately ^41^. To regenerate skeletal muscle, recent studies have explored the use of ASC-derived EVs ^17, 42^ and MSC-derived exosomes ^15^ injected at various time points at the site of injury or intravenously. These studies also measured various outcomes from an increase in cross-sectional area of newly formed fibers ^17^ to increased regulation/expression of myogenic genes ^42^, capillary density, myofiber diameter, number of centrally located nuclei, and decreases in fibrotic area ^15^.

## Recent advances in understanding immunomodulatory roles of stem cells

The immunomodulatory effects of MSCs have been studied for over two decades. MSCs regulate immune cell activity via direct cellular contact as well as cytokine and growth factor secretion. Recently, MSCs were shown to negatively regulate the activation and proliferation of T cells during injury and to enhance the immunosuppressive capacity of regulatory T cells in culture ^43^. Conversely, innate immune cells exert an effect on stem cells in musculoskeletal tissues, as demonstrated by the induction of calvarial osteoblast mineralization by macrophages in 2D culture, the induction of osteogenesis in MSCs grown in media from IL-4–stimulated macrophages ^44^, and the link between macrophage activation and pro-osteogenic gene expression in MSCs in 3D culture ^43^. A further understanding of the crosstalk between implanted stem cells and immune cells will help us to maximize the regenerative capacity of stem cells in musculoskeletal therapy.

## Advances in imaging technologies may provide insights into stem cell fate

Recent imaging advances, including novel optical clearing techniques ^45, 46^ combined with light sheet microscopy ^46^ and quantitative confocal microscopy ^47^, have enabled the spatial mapping of endogenous stem cells in their native 3D environment within musculoskeletal tissues and the monitoring of stem cell location and viability after implantation. These advances in imaging have been applied to the visualization of the bone marrow cavity in whole mouse femurs ^45^, the quantification of the abundance of cell populations previously underestimated by standard flow cytometry, and the definition of subpopulations on the basis of location and morphology ^47^. Other advances in magnetic resonance imaging ^48^, bioluminescence ^49, 50^, photoacoustic imaging ^51^, ultrasound, and magnetic particle imaging ^52^ may also be applied to the *in vivo* visualization of implanted stem cells.

## Concluding statements

Future advances toward the clinical application of stem cells for musculoskeletal treatments will require that tissue engineering studies move beyond empirical readouts and employ more rigorous tools to identify the molecular mechanisms underlying regenerative outcomes. Specifically, there needs to be a greater emphasis on the development and use of novel imaging techniques (to spatially map transplanted and endogenous stem cells and immune cells in tissue-engineered grafts post-implantation to visualize cell fates and interactions) as well as on coupling these data with single-cell analytics. The combined application of these advanced molecular tools will enable further insight into the actual role that stem cells are playing and will facilitate better targeting and optimization of their use in promoting tissue regeneration.
